# The basidiomycetous yeast *Trichosporon* may cause severe lung exacerbation in cystic fibrosis patients – clinical analysis of *Trichosporon* positive patients in a Munich cohort

**DOI:** 10.1186/1471-2466-13-61

**Published:** 2013-11-01

**Authors:** Carolin Kröner, Matthias Kappler, Ann-Christin Grimmelt, Gudrun Laniado, Benjamin Würstl, Matthias Griese

**Affiliations:** 1 Pediatric Pneumology and Christiane Herzog Cystic Fibrosis Center, Dr. von Hauner Children’s Hospital, Ludwig-Maximilians University, Lindwurmstr. 4, 80337 Munich, Germany; 2 Max von Pettenkofer Institute, Ludwig-Maximilians University, Pettenkoferstr. 9a, 80336 Munich, Germany

**Keywords:** Cystic fibrosis, *Trichosporon* species, Respiratory pathogens, Exacerbations

## Abstract

**Background:**

The relevance of *Trichosporon* species for cystic fibrosis (CF) patients has not yet been extensively investigated.

**Methods:**

The clinical course of CF patients with *Trichosporon spp.* in their respiratory secretions was analysed between 2003 and 2010 in the Munich CF center. All respiratory samples of 360 CF patients (0 – 52.4 years; mean FEV1 2010 81.4% pred) were investigated.

**Results:**

In 8 patients (2.2%, 3 male, mean age 21.8 years) *Trichosporon* was detected at least once. One patient carried *T. asahii.* One patient carried *T. mycotoxinivorans* and one patient *T. inkin* as determined by DNA sequencing. As potential risk factors for *Trichosporon* colonization steroid treatment, allergic bronchopulmonary aspergillosis (ABPA) and CF associated diabetes were identified in 6, 5, and 2 patients respectively. For one patient, the observation period was not long enough to determine the clinical course. One patient had only a single positive specimen and exhibited a stable clinical course determined by change in forced expiratory volume in one second (FEV1), body-mass-index (BMI), C-reactive protein (CRP) and immunoglobulin G (IgG). Of 6 patients with repeatedly positive specimen (mean detection period 4.5 years), 4 patients had a greater decline in FEV1 than expected, 2 of these a decline in BMI and 1 an increase in IgG above the reference range. 2 patients received antimycotic treatment: one patient with a tormenting dry cough subjectively improved under Amphotericin B inhalation; one patient with a severe exacerbation due to *T. inkin* was treated with i.v. Amphotericin B, oral Voriconazole and Posaconazole which stabilized the clinical condition.

**Conclusions:**

This study demonstrates the potential association of *Trichosporon spp.* with severe exacerbations in CF patients.

## Background

*Trichosporon* species (*T. spp.*) belong to the genus of basidiomycetous yeast and are widely distributed in nature. They are found in soil and water and are known to colonize skin and gastrointestinal tract of humans [1,2]. Numerous *Trichosporon spp.* have been characterized [3], while only a few cause human disease including *T. asahii, T. inkin, T. asteroides, T. cutaneum, T. mucoides, T. ovoides* (formerly subsumed as a single species: *T. beigelii*[4,5]) and two more recently described species: *T. louberi* and *japonicum*[6]. The most relevant disease entities caused by *Trichosporon spp.* are white piedra, a mycosis of the hair (*T. inkin, T. ovoides*) [4], summer-type hypersensitivity pneumonitis in Japan (*T. asahii, T. mucoides* and *T. dermatis*) [4] and invasive trichosporonosis (local and disseminated; most often caused by *T. asahii*) [4]. Risk factors for invasive trichosporonosis are immunosuppression, including prolonged corticosteroid treatment, impaired granulocyte function, neutropenia, malignancies, especially leukemia [7], renal disease, extensive burns and HIV [7,8]. Invasive Trichosporon infections are the second most common yeast fungaemia in humans [4,9], especially in patients with haematological malignancies [10]. Symptoms of invasive trichosporonosis range from pulmonary infiltrates, skin lesions and renal failure to local organ infections, such as hepatitis [4].

The significance of *Trichosporon spp.* for CF patients has so far not been widely recognized. Only three case reports are available in literature: One 11-year old CF patient suffered from a non-aspergillus allergic bronchopulmonary mycosis due to *T. beigelii* and recovered within 2 months under intensive steroid and antifungal therapy [11]. One 20-year old, male CF patient died within a few days from admission due to a fulminant pneumonia with *T. mycotoxinivorans*[12] and one 35-year old CF patient died due to a disseminated fatal infection with *T. mycotoxinivorans* 29 days after lung transplantation [13]. Aim of this study was to investigate the detection rate of *Trichosporon spp.* in a German CF cohort and to describe the clinical course of patients positive for *Trichosporon*.

## Methods

All respiratory samples of 360 CF patients (mean age 2010 18.11 years, range 0 – 52.4; mean FEV1 81.4% pred; range 16 – 136% pred; 12.5% with associated diabetes mellitus) who attended the CF Center at the Children’s University Hospital in Munich were studied between January 1st, 2003 and December 31st, 2010 for the presence of bacterial and/or fungal pathogens. Other detected fungi included *Aspergillus* species (approximately 16% of the cohort, >90% *Aspergillus fumigatus*) and *Candida species* (approximately 40% of the cohort, >90% *Candida albicans*). 15% of the patients exhibited intermittent *Pseudomonas aeruginosa* colonization, 48% chronic colonization. Patient visits took place on average every three months, if indicated more often. At each visit, sputum samples or throat smears were taken and the patients were investigated clinically; routine laboratory examinations including inflammatory parameters and lung-function tests were performed. On average 4 respiratory samples/patient/year were analysed, approximately 70% of the samples were throat swabs, 30% expectorated sputum. The clinical course of *Trichosporon* positive patients was analysed retrospectively based on forced expiratory volume in one second (FEV1,%pred), body mass index (BMI) and the inflammatory markers CRP (mg/l) and immunoglobulin G (IgG, g/l), the latter known to be associated with chronic inflammation and inversely correlated with lung function and long-term prognosis [14,15]. The erythrocyte sedimentation rate 1 h (ESR, mm) was also recorded; however, insufficient data was available for analysis. Decline in FEV1 was defined as a drop of the yearly mean FEV1 of >1%pred/year from first detection to the end of the observation period of the individual patient, based on the average yearly drop of FEV1 values of 1% in large CF registries [16]. Any change in BMI was recorded, increase in CRP was defined as an increase by >5 mg/l and an increase in IgG was defined as an increase by >3 g/l from first detection to the end of the observation period. Informed consent was obtained from all participants and the study was approved by the institutional review board of the University of Munich.

### Specimen characterization

Microbiological examinations were routinely performed at the department of microbiology at the Dr. von Hauner Children’s hospital in Munich. Specimen with newly identified microorganisms were also analysed at the Max von Pettenkofer-Institute in Munich (National Consiliary Laboratory for CF Bacteriology, South Germany). Clinical specimens were diluted 1:2 with Dithiothreitol (DTT; Sigma, Deisenhofen, Germany) solution (1 mg/ml) for liquefaction. For quantitative bacteriology sputum samples were diluted 10^-1^, 10^-4^ and 10^-5^ with 0,9% NaCl and plated on Trypticase soy agar (TSA) and McConkey Agar. DTT-pretreated samples were further streaked on Columbia Agar with 5% sheep blood, Chocolate agar, Sabouraud-Glucose-Agar for fungi as well as on selective media containing either 8 μg/ml Meropenem or 16 μg/ml Polymyxin B to recover Carbapenem-resistent gram-negative bacteria and *Burkholderia cepacia* complex isolates, respectively. Columbia Agar and Chocolate agar were incubated at 32°C under anaerobic conditions to prevent overgrowth of clinically important bacteria such as *H. influenza* by *P. aeruginosa*, while all other media were incubated aerobically at 32°C. All bacteriological cultures were incubated for 3–5 days and Sabouraud-Glucose-Agar for at least 10 days. Species identification was performed by routine microbiological procedures. Yeasts were isolated on chromogenic Agar plates for differentiation. Filamentous fungi were primarily differentiated by micromorphological examinations.

*Trichosporon spp*. in pts 1,2,4,6,7,8 were identified by alternative biochemical test (API 20 C AUX and ID 32 C galleries bioMérieux, Mercy l’Etoile, France). *T. asahii* (patient 2) was identified by microscopic identification, biochemical testing and Matrix Assisted Laser Desorption Ionisation – Time of flight Mass Spectrometry (MALDI-TOF MS Biotyper system Bruker Daltonics). Three strains (two strains of patient 3, one strain of patient 5) were further analysed - prompted by the clinical deterioration of these patients after *Trichosporon* detection. In a first step these strains were identified by the MALDI-TOF MS BioTyper system; in a second step DNA sequencing was performed to confirm the MALDI-TOF results. MALDI-TOF MS is a reliable method to rapidly identify fungal species, and can be used as an alternative to PCR-based techniques [17,18], which have been extensively used for the identification of *Trichosporon spp.*[19]. DNA sequencing of conserved (D1/D2 region of the 28S rDNA) and variable regions (ITS and IGS1 regions) are common methods to determine *Trichosporon spp.*[20,21].

**Figure 1:**
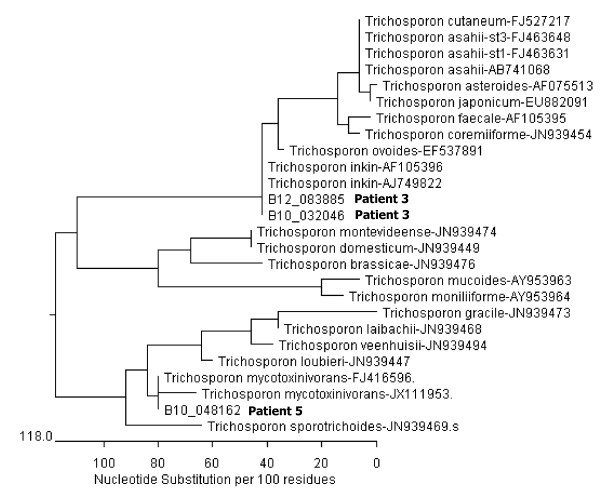
**Phylogenetic tree of *Trichosporon spp *(28S rDNA).** Discrimination between the sequenced isolates of patient 3 (one isolate from 2010 and one from 2012) and of patient 5 and the most common *Trichosporon spp* (NCBI Blast). A comparable sequence length of 562 bp was used to realize alignment, performed by MegAlign, software version 8.1.4, Lasergene, DNASTAR. The bar shown in the graph provides scale to the branch lengths and represents the number of substitutions per 100 residues.

In this study the detected mass spectra in the MALDI-TOF analysis of the two patients 3 and 5 (scores >1.700) were compared to the reference spectra of *Trichosporon spp.* included in the MALDO Biotyper BDAL MSP library. The results were confirmed by DNA sequencing: Parts of the 28S rDNA were sequenced and compared with fungal consensus sequences of *Trichosporon spp.* in the NCBI BLAST^®^. Parts of the 28S rDNA region were amplified by PCR using the following oligonucleotide primers: primer P1( 45–64): 5’-ATCAATAAGCGGAGGAAAAG-3’ and primer P2 (825–843): 5’-CTCTGGCTTCACCCTATTC-3’. The identity of sequenced strains was 99.0% for *T. inkin* and *T. mycotoxinivorans* respectively. Figure 1 demonstrates the discrimination between the sequenced isolates and the most common *Trichosporon spp*. Due to varying lengths of published sequences (500 to 1000 bp) a comparable sequence length of 562 bp was used to realize the alignment performed by MegAlign, software version 8.1.4, Lasergene, DNASTAR^®^.

## Results

*Trichosporon spp.* were detected in 8 of 360 patients (i.e. 2.2%; 3 male; mean age: 21.8 years, range: 8 – 41.1; Table 1). Two *Trichosporon spp.* were specified by MALDI-TOF MS and by DNA sequencing: *T. mycotoxinivorans* and *T. inkin*. *T. asahii* was identified by microscopy, biochemical testing and MALDI-TOF MS. All but one patient were colonized with further CF typical microorganisms at first detection as depicted in Table 1. 5 patients had a history of ABPA 0–5 years prior to first detection. 5 patients had a history of treatment with nebulized steroids and/or steroid inhalers, 3 of whom had received additional systemic steroid treatment, one patient had received systemic steroid treatment only. 2 patients had CF associated diabetes at first detection. No patient underwent lung transplantation. All 8 patients had a dF508 mutation, 3 patients an additional stop mutation (2 patients R553X and one patient S466X) (Table 1).

**Table 1:** **Characterization and follow-up of CF patients positive for *Trichosporon***

**Patient number**	**1**	**2**	**3**	**4**	**5**	**6**	**7**	**8**
**Sex**	F	M	F	M	F	F	M	F
**Mutation**	dF508/ dF508	dF508/ dF508	dF508/ Nk	dF508/ R553X	dF508/ S466X	dF508/ Nk	dF508/ Nk	dF508/ R553X
**Age at first detection (y)**	41.1	33.0	10.0	11.0	21.7	8.0	22.3	27.6
**Age at last detection (y)**	43.0	36.9	17.8	11.0	28.4	8.6	29.2	28.4
Follow up (y)^*^	3.1	5.9	7.8	2.4	6.7	5.3	6.9	0.8
***Trichosporon *** **species**	Nk	Asahii	Inkin	Nk	Mycotoxinivorans	Nk	Nk	Nk
**Other isolated organisms from sputum (at first detection)**	Ps ae (chron), Ps ae muc	E col, Prot mirab	None	Norcard farc, Inqu lin, Burc cep, Asp fum, Penicill sp	Ps ae (chron), Cand alb	Acinet baum, fungi (not spec), Ps ae (chron)	Ps ae (chron), Ps ae muc, Cand alb	Ps ae muc (chron), Cand alb
**Other isolated organisms from sputum (throughout observation period)**	Pseudoall boyd (rep), Cand glab (rep), Cand alb (rep), Cand paraps (rep), Enteroc spp. (rep), Asp fum (rep)	Prot mirab (rep), Asp fum (rep)	Asp fum (rep), Sten mal (rep), Cand alb (rep), Sa (rep)	Inqu lin (rep), Burc cep (rep), Penicill sp (rep), Acinet fum (rep), Ps put (once)	E coli (rep), Sten malt (rep), Asp flav (rep)	Acinet baum (rep), Pet sord (rep)	Asp fum (rep), Prot mirab (rep), Sa (rep), Enteroc spp (rep), Cand alb (rep)	Cand paraps (rep)
**Antibiotic regimen prior to first detection (agent; regimen)**								
**Oral**	Cip (cont)	Cefur (cont), Cip (2 w on/off)	Cep (cont), Cip (2 w on/off)	Ceph (cont), Cip (2 w on/off)	Ceph (cont)	Ceph (cont), cip (2 w on/off)	Ceph (cont)	Cot (cont)
**Inhaled**	Tob/col (2 w on/off)	None	Col (2 w on/off)	Tob/col (2 w on/off)	Gern (2 w on/off)	None	Gern (2 w on/off)	Gern/col (2 w on/off)
**iv (courses/y)**	2	2	1	1	2	1	1	2
**Diabetes mellitus (at first detection)**	Yes	Yes	no	No	No	No	No	No
**ABPA (years prior to first detection)**	5y, 2y	3y	no	3y, 1y	No	1 y after first detection	No	At first detection
**Nebulized/inhaled steroids prior to first detection (duration)**	Yes (5y, rep)	No	No	Yes (2.5y)	Yes (2y)	Yes (1y)	No	Yes (from first detection on)
**Intermittent systemic steroids prior to first detection**	Yes	Yes	No	Yes	Yes	No	No	Yes (from first detection on)
Lung function FEV 1 (% pred)^**^	Decline	Stable	Decline	Increase	Decline	Increase	Decline	Np
**At first detection**	61.2	49.3	81.2	84.3	86.3	68.2	40.1	61.3
**At end of observation**	43.1	47.2	57.3	88.3	79.0	79.6	30.0	Np
**BMI (kg/m**^**2**^)^***^	Decline	Stable	Increase	Increase	Increase	Increase	Decline	Np
**At first detection**	18.9	25.8	13.7	15.5	19.6	15.1	19.3	21.5
**At end of observation**	18.2	25.8	17.8	16.3	19.9	18	18.5	Np
IgG (g/l)^****^	Increase^+^	Stable	increase	Stable	Stable	Stable	Increase	Np
**At first detection**	18.8	13.0	8.1	8.2	10.4	9.5	13.4	9.2
**At end of observation**	22.4	13.7	13.1	10.2	11.7	9.6	17.1	Np
CRP (mg/l)*****	Increase	Na	Increase	Stable	Stable	Stable	Decrease	Np
**At first detection**	6.2	11.1	<1.0	<1.0	3.5	<1.0	19.9	17.9
**At end of observation**	30.7	Na	11.7	<1.0	5.4	4.1	6.7	Np

*years from first detection to Trichosporon to end of observation period, **change throughout the observation period of the individual patient, decline defined as ΔFEV1 > 1%pred/year, ***change in BMI throughout the observation period, ****change throughout the observation period, change defined as ΔIgG >3 g/l,, *****change throughout the observation period, change defined as ΔCRP >5 mg/l, + above reference range.

*Abbreviations*: *Na* = not available, *Nk* = not known, *np* = not possible, *chron* = chronic, *cont* = continuous, *rep* = repeated, / = alternating, *w* = weeks, *y* = years, *Acinet baum* = Acinetobacter baumannii, *Acinet fum* = Acinetobacter fumigatus, *Asp flav* = Aspergillis flavipes, *Asp fum* = Aspergillus fumigatus, *Burc cep* = Burkholderia cepacia, *Cand alb* = Candida albicans, *Cand glab* = Candida glabrata, *Cand paraps* = Candida parapsilosis, *Cefu* = cefuroxim, *Cep* = ceporexin, *Ceph* = cephalexin, *Cip* = ciprofloxacin, *Col* = colistin, *Cot* = cotrimoxazole, *E coli* = Escherichia coli, *Gern* = gernebcin, *Inqu lin* = Inquilinus limosus, *Nocard farc* = Norcarderia facrinica, *Pet sord* = Petriella sordida, *Penicill Sp* = Penicillium sp, *Prot mirab* = Proteus mirabilis, *Pseudoall boyd* = Pseudoallescheria boydii, *Ps ae* = Pseudomonas aeruginosa, *Ps ae muc* = Pseudomonas aeruginosa mucoides, *Ps put* = Pseudomonas putida, *Sa* = Staphylococcus aureus, *Sten malt* = Stenotrophomonas maltophilia, *Tob* = tobramycin.

### Clinical course of patients positive for *Trichosporon*

**Figure 2:**
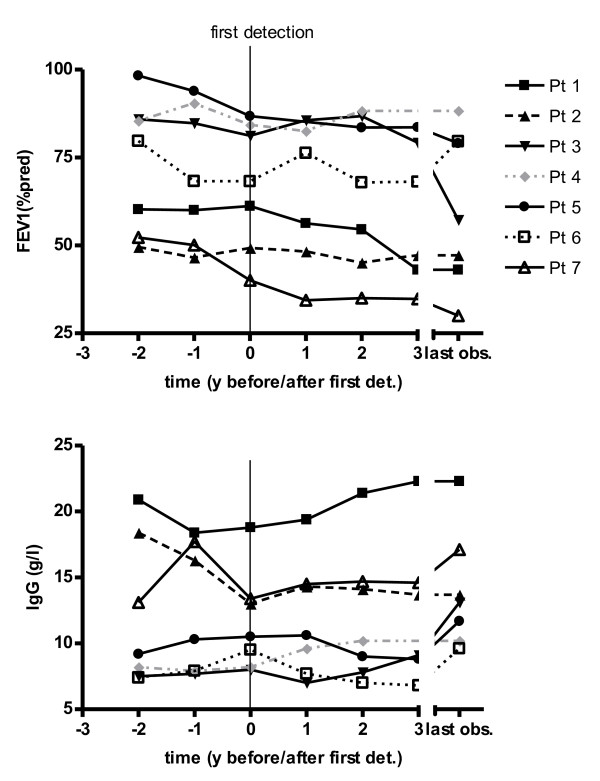
**Course of FEV1 (%pred) and the inflammatory marker IgG (g/l) of CF patients positive for *Trichosporon*.** Mean FEV1 and IgG values for 7 *Trichosporon* positive patients with sufficiently long follow-up periods at 7 time intervals: 1 and 2 years before first *Trichosporon* detection, at first detection, 1, 2 and 3 years after first detection and at the end of the observation period. The mean number of data points per year was 4 (range 2–7). Each symbol/line represents one patient. The solid lines represent the patients with a decline in FEV1. The grey line represents the patient with only a single *Trichosporon* positive specimen.

The observation period for patient 8 was 0.8 years and thus not long enough for an analysis of the clinical course. One patient (4) had only a single positive respiratory specimen and exhibited a stable FEV1, stable CRP, IgG and an increase in BMI. The other 6 patients (1,2,3,5,6,7) had repeatedly *Trichosporon* positive specimens with a mean detection period of 4.5 years (range 0.6 – 7.1). FEV1, BMI, CRP and IgG remained stable in 1, respectively 2 of these patients (2,6) with a mean detection period of 2.5 years (range 0.6 – 3.9). FEV1 declined by a mean ΔFEV1 of – 2.9pred%/year (range -1.1pred%/year to -5.8pred%/year) in 4 patients (1,3,5,7) with repeated positive samples and a mean detection period of 5.7 years (range 1.9-7.1). CRP increased in 2 of these patients (1,3) and IgG increased for 3 of these patients (1, 3, 7), in patient 1 above the reference range. BMI declined in 2 (1,7) of these patients (Table 1, Figure 2).

### Treatment

Patients 3 and 5 received antimycotic treatment due to an unexplained but marked deterioration of the clinical status. Patient 5 with a prolonged detection period of *T. mycotoxinivorans* exhibited a recurrent and tormenting dry cough; she reported a substantial subjective improvement of the dry cough after inhalation with nebulized Amphotericin B (not liposomal, 15 mg 1:1 diluted with distilled water, 1x/day for 4–8 weeks, 3x/year). *T. mycotoxinvorans* however persisted in the sputum. Patient 3 is described in detail below.

### Clinical course of patient 3

**Figure 3:**
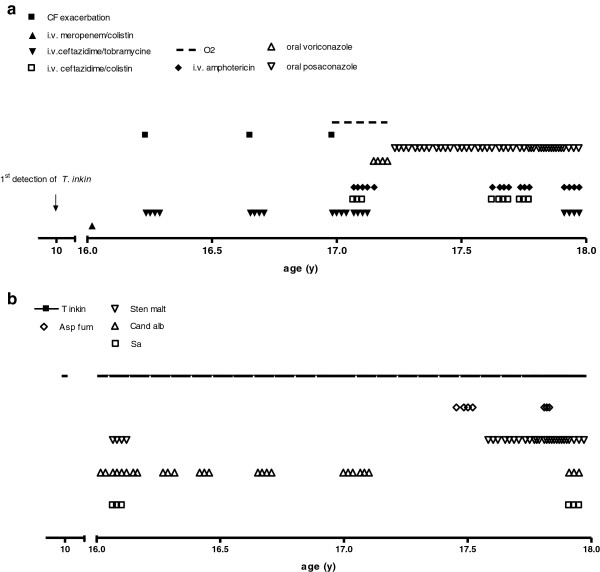
**Clinical course and colonization with other microorganisms of a patient (3) infected with *T. inkin. *a**: Clinical course: time-points of exacerbations and therapeutic interventions. **b**: Colonization with different respiratory microorganisms.

**Figure 4:**
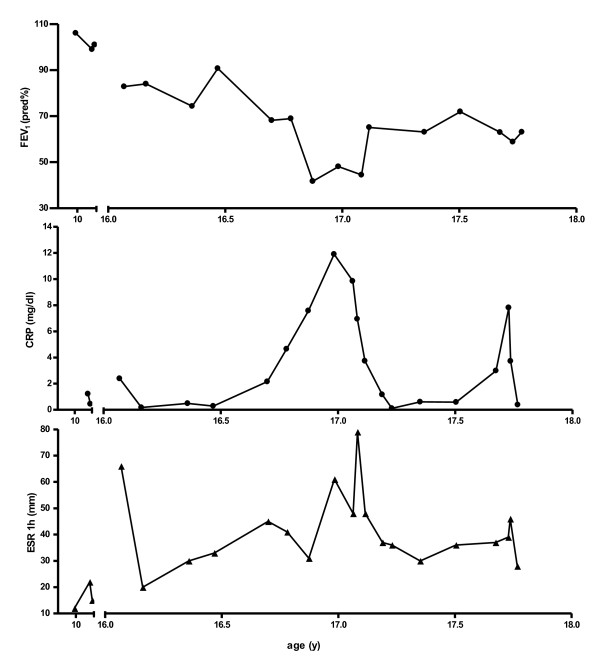
**Course of FEV1 (% pred), CRP (mg/dl) and ESR 1 h (mm) of a patient (3) infected with *T. inkin***

**Figure 5:**
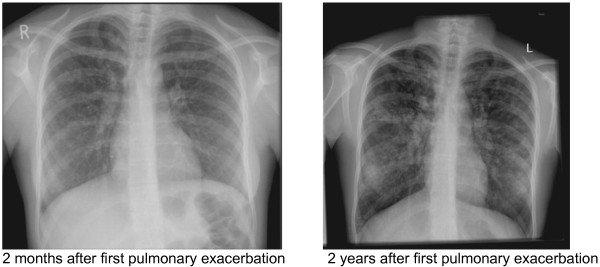
**Chest X-ray of a patient (3) infected with *T. inkin *two months and two years after first pulmonary exacerbation due to *T. inkin*.** The second X-ray demonstrates a strong increase in hyperinflation, increasing bronchiectasis and mucus plugging, increasingly thickened bronchial walls and numerous additional patchy lesions.

Until the age of 16 years a female CF patient (ΔF508/not known) with pancreatic insufficiency had only minor respiratory symptoms, FEV1 values between 85 and 106 pred% and a BMI between the 3rd to 5th percentile. *Trichosporon spp.* was detected for the first time at the age of 10 years (Figure 3a). Further respiratory pathogens at first detection of *Trichosporon* included *Stenotrophomonas maltophilia, Staphylococcus aureus, Candida albicans and Aspergillus fumigatus* (Figure 3b). The patient was treated with standard CF therapy and had no history of systemic or nebulized steroids. At the age of 16 years, the stable clinical course was disrupted by 4 severe exacerbations within 12 months requiring intensive treatment (Figure 3a). The patient exhibited dyspnea, bloody sputum, an oxygen saturation of 89% in ambient air, night sweats, and a weight loss of 5 kg. FEV1 dropped to 45 pred% (Figure 4). Due to the lack of response to intensive i.v. antibiotic and supportive treatment in the presence of *Trichosporon inkin*, i.v. antimycotic treatment with liposomal Amphotericin B (4.6 mg/kg/d) was initiated; nebulized Amphotericin B was not tolerated. Resistance testing of *Trichosporon inkin* revealed good effectiveness of Amphotericin B, Posaconazole and Voriconazole. After 8 weeks of Amphotericin treatment, the patient’s condition improved considerably and oral therapy with Voriconazole was initiated. FEV1 had increased from 45 pred% to 65 pred% (Figure 4). The patient developed an impaired color vision and Voriconazole was replaced with Posaconazole, which has been continued since. 12 months later 3 more episodes of clinical deterioration had been treated with i.v antibiotics and i.v. Amphotericin B. The clinical status stabilized with FEV1 values between 55 and 70 pred%. The chest x-rays taken 2 months and 2 years after the first exacerbation show the extent of damage (Figure 5). *Trichosporon inkin* persists in the sputum until the current age of 17.8 years.

## Discussion

The relevance of fungal colonization in CF patients has only recently been recognized [22]. Colonization with *C. albicans* and *A. fumigatus* –also in non-ABPA patients- has for example been associated with a decline in lung function and a negative effect on the clinical outcome [23,24]. *A. fumigatus* in particular was demonstrated to have a pro-inflammatory effect on the CF epithelium [25]. However, the available studies are limited by important confounders, such as the lack of standardized detection methods or co-infection with other microorganisms. The contribution of fungi to CF lung disease thus remains controversial as does the indication to a non-risk free antifungal therapy.

The prevalence and the relevance of *Trichosporon spp.* in CF has to date not been investigated. In the present study *Trichosporon spp.* was found in respiratory samples of 2.2% of the cohort over an eight year period. Colonization rates of *Trichosporon spp.* in non-CF patients range between 0.8% in throat cultures and 3.1% in stool cultures of patients in a large Veterans hospital [26] and 3.7% in stool, skin or urine of 317 immunosuppressed hosts [27].

Risk factors for colonization and/or infection with *Trichosporon* in CF are also unknown. Invasive trichosporonosis in non-CF patients has been associated with mucosal disruption and general immune dysfunction due to HIV or malignancies [28], however, the exact pathophysiology of *Trichosporon* infections is unknown. It is possible that mucosal disruption in CF also predisposes for *Trichosporon* colonization. Our data suggest that age, prior systemic or inhaled steroid treatment, ABPA and possibly CF associated diabetes might predispose for *Trichosporon* colonization. 3 of 8 patients carried a stop mutation in addition to dF508, which is higher than the general frequency of stop mutations in German CF population (1.8% for R553X; <1% for S466X) [29]. Our data further indicate that longer *Trichosporon* detection periods may be associated with a decline in lung function and BMI, and increased inflammation. Chronic airway colonization with *Trichosporon* may harbour the risk of clinically relevant infection leading to unstable lung disease. Further studies will have to corroborate these findings.

Limitations of the current study include the relatively small amount of patients, the retrospective design, the presence of other microorganisms as a confounder and the lack of specification of all *Trichosporon spp*. Standardized diagnostic procedures were subsequently introduced.

Up to now three case reports of CF patients have demonstrated the potential of exacerbation and in two cases of a fatal, disseminated infection due to *Trichosporon spp.*[11-13]. Here we add another patient (3) whose course was significantly affected by infection with *T. inkin*. This conclusion is based on the unexplained failure of intensive antibacterial and supportive therapy and the clear improvement and stabilization of the clinical status after the initiation of antifugal therapy. Repeated detection of *Trichosporon spp.* may indicate CF patients at risk for deterioration; preemptive treatment may be considered.

## Conclusion

To conclude, emerging pathogens relevant for CF lung disease may include the basidiomycetous yeast *Trichosporon*. Its detection in respiratory secretions in patients not responding to standard antimicrobial therapy may warrant targeted antifungal therapy.

## Competing interests

The authors declare that they have no competing interests.

## Authors’ contributions

MG, and CK designed the study. MG and CK collected the data and drafted the manuscript. MK and AC participated in the design and coordination of the study and helped to draft the manuscript. BM and GL performed the microbiological studies. All authors read and approved the final manuscript.

## Pre-publication history

The pre-publication history for this paper can be accessed here:

http://www.biomedcentral.com/1471-2466/13/61/prepub
